# Association between Gastric Cancer Risk and Serum *Helicobacter pylori* Antibody Titers

**DOI:** 10.1155/2017/1286198

**Published:** 2017-06-11

**Authors:** Mitsutaka Shuto, Toshio Fujioka, Osamu Matsunari, Kazuhisa Okamoto, Kazuhiro Mizukami, Tadayoshi Okimoto, Masaaki Kodama, Shigeru Takigami, Chuichi Seguchi, Yoshihito Nonaka, Ryugo Sato, Yoshio Yamaoka, Kazunari Murakami

**Affiliations:** ^1^Department of Gastroenterology, Oita University Faculty of Medicine, Oita, Japan; ^2^Takada Chuo Hospital, Oita, Japan; ^3^The Bungotakada City Medical Association, Oita, Japan; ^4^Oita Kouseiren Tsurumi Hospital, Oita, Japan; ^5^Department of Environmental and Preventive Medicine, Oita University Faculty of Medicine, Oita, Japan

## Abstract

**Background/Aims:**

It is difficult to confirm the accurate cutoff value to diagnose *Helicobacter pylori (Hp)* infection using commercial serology kits. It is reported that there were many cases with present/past infection that even the serum *Hp*-IgG antibody (*Hp*Ab) titers were below the cutoff value (e.g., 10 U/mL for E-Plate®), suggesting that we might overlook many gastric cancer (GC). We investigated an association between gastric cancer risk and serum *Helicobacter pylori* antibody titers.

**Methods:**

We conducted a primary screening between 2014 and 2015. We performed gastroendoscopy if *Hp*Ab titers were ≥3.0 U/mL (i.e., more than measurable limit, E-Plate). These patients were divided into two groups: *Hp*Ab = 3.0–9.9 U/mL (“negative-high” group) and *Hp*Ab ≥ 10 U/mL; cutoff value (“over-10 U/mL” group). *Hp* infection status was investigated, and the number of GC patients was counted.

**Results:**

Among the 3321 subjects in the primary screening, 56.9% (1891/3321) showed *Hp*Ab titers ≥3.0 U/mL; 1314 patients underwent gastroendoscopy. Ten were GC. 421 patients were “negative-high” group; two were GC. After evaluating 381 patients for *Hp* infection, 22.6%/60.6% was with present/past infection among the “negative-high” group.

**Conclusion:**

We also found a correlation between *Hp*Ab titers and *Hp* infection status. “Negative-high” group has a risk of GC.

## 1. Introduction

*Helicobacter pylori* (*Hp*) infection and *Hp*-related atrophic gastritis are regarded as risk factors for gastric cancer (GC) [1–8]. It has been reported that the risk of GC is very low in *Hp*-uninfected patients, but is high in patients with a present or past *Hp* infection [2]. The International Agency for Research on Cancer (IARC Working Group) of the World Health Organization (WHO) reported that as of September 2014, about 80% of GC throughout the world was associated with *Hp* infection and that *Hp* eradication therapy could reduce the incidence of GC by 30–40% [1].

Testing to diagnose *Hp* infection includes the rapid urease test, histological analysis using microscopy, and cultures, which reflect *Hp* infection at the biopsy site, and the urea breath test, serum *Hp*-IgG antibody (*Hp*Ab) titers, and stool antigen tests, which reflect *Hp* infection in the entire stomach [9, 10]. Serum *Hp*Ab testing is an indirect method for diagnosing *Hp* infection by measuring antibodies as a localized immune reaction in the gastric mucosa to *Hp* infection. However, *Hp*Ab titers decrease after a successful eradication therapy [11–14], and because the rate of the decrease and the amount of change in titers vary among individual patients, these titers are unsuitable to evaluate the cure of infection and not useful in management of *Hp* infection [15–18]. Moreover, *Hp*Ab titers may not be positive just after *Hp* infection or with immune dysfunction; spontaneous clearance of *Hp* with severe atrophic gastritis or unexpected eradication of *Hp* due to antibiotics also can lead to negative results [12, 19]. Several recent studies have reported an association between serum *Hp*Ab titers and GC risk [20–24].

Interestingly, Tatemichi et al. [21] performed a prospective study and reported that the risk for developing GC was even higher in atrophic gastritis patients with low serum *Hp*Ab titers close to 10 U/mL than those with high *Hp*Ab titers.

In Japan E-Plate, a commercial serology kit, produced by Eiken Chemical Co. Ltd, Tokyo, Japan, is the most commonly used. This kit is a direct enzyme immunoassay (EIA) kit, and the cutoff value instructed by the kit is 10 U/mL, and the value has been widely applied to large studies analyzing Japanese participants [19–21]. However, there are increasing evidence that some patients have a present or past *Hp* infection and GC risk despite *Hp*Ab titers <10 U/mL [25]. Patients recently infected with *Hp*, who have had *Hp* eradication therapy, or who have had spontaneous clearance of *Hp*, may have a false negative diagnosis of infection, and thus the risk of GC may be overlooked. Therefore, recently, the Japanese Society for Helicobacter Research (JSHR) stated that “a considerable number of patients with serum *Hp*Ab titers below the cut-off value have a present or past *Hp* infection; therefore we caution against assuming there is no GC risk” [25].

It will be difficult to establish an optimal cutoff value of *Hp*Ab titers, and we hypothesized that patients with any measurable *Hp*Ab titers (≥3.0 U/mL in a case of E-Plate) might have a risk of GC. Therefore, we investigated an association between gastric cancer risk and serum *Helicobacter pylori* antibody titers.

## 2. Materials and Methods

The Bungotakada city had carried out GC examination program using X-ray contrast studies based on the guidance of the Japanese government; however, the consultation rate was extremely low. Since the top cause of death among male with late middle age at the city was GC, we conducted the GC risk screening program focused on the *Hp* as the most important risk factor of GC in cooperation with the city.

The *Hp*Ab titers were measured at 80 venues prepared for health checkup or any hospitals/clinics in the city, and the examination fees were completely covered by budgets of the city. We also performed an open lecture for citizen to teach the knowledge of GC, especially focused on the importance of *Hp* examination (280 citizens were participated). There are 145 community associations in the city, and we also explained the importance of the study to the association members. Furthermore, we used the local cable TV twice to make an overture for participating the study.

This study was conducted as a primary screening in cooperation with Bungotakada city in residents age ≥ 20 years between April 1, 2014 and March 31, 2015. Among a total population of 23,244 (as of August 11, 2016), 3321 subjects (14.3%) participated the study. Among subjects aged 65 to 74 years old, 35.1% of population participated, followed by 31% for those aged 75 years or older, and 30.8% for those aged 40 to 64 years old. In contrast, only 3.1% of the population aged 20 to 39 years old could participate the study. Primary screening included blood tests for measurement of *Hp*Ab. Measurements were performed using an E-Plate (Eiken Chemical Co. Ltd., Tokyo, Japan).

The cutoff value instructed by the kit is 10 U/mL; however, in this study, *Hp*Ab titers of 3.0 U/mL (i.e., more than the measurable limit) were used as the cutoff value for primary screening, with *Hp*Ab ≥ 3.0 U/mL considered positive. Positive patients who could obtain the informed consent underwent gastroendoscopy as a secondary screening at a medical service under the coverage of national health insurance (inclusion criteria). In addition to detailed examination by gastroendoscopy for GC, mucosal atrophy, an important finding in *Hp*-related gastritis, and *Hp* infection status were evaluated.

Gastric mucosal atrophy was assessed by the Kimura-Takemoto classification system [26] for patients who underwent gastroendoscopy at Takada-Chuo Hospital. Mucosal atrophy is classified as C-1: limited to the antrum, C-2: limited to the angle, or C-3: extending into the upper corpus. Further classification includes O-1: atrophy reaching to the cardia, but with preservation of the greater curvature fold, O-3: atrophy of the entire stomach, and O-2: intermediate between O-1 and O-3. In this study, gastric atrophy was defined as C-2 or greater.

*Hp* infection status was evaluated at Takada-Chuo Hospital with at least one of the following: rapid urease test, urea breath test, or stool antigen test. A PyloriTek® Test Kit (Sakura Finetek Japan Co. Ltd., Tokyo, Japan) was used for the rapid urease test, POCone™ (Otsuka Electronics Co. Ltd., Tokyo, Japan) was used for the urea breath test, and Testmate Pylori Antigen EIA (Kyowa Medex Co. Ltd., Tokyo, Japan) was used for the stool antigen test. PPI were discontinued at least two weeks before testing for *Hp* infection.

Positive results with the above tests were regarded as a “present infection.” Negative results with the above tests, but those with the presence of endoscopic atrophy, were regarded as a “past infection.” Negative results with the above tests and the absence of endoscopic atrophy were regarded as “uninfected.”

Patients with *Hp*Ab 3.0–9.9 U/mL were defined as a “negative-high” group. Secondary screening patients were divided into two groups, the “negative-high” group (*Hp*Ab 3.0–9.9 U/mL) and the “over-10 U/mL” group (*Hp*Ab ≥ 10 U/mL). Mean age, sex, number of patients with GC, severity of endoscopic atrophy, and *Hp* infection status were compared.

Statistical analysis was performed using the chi-square test. The level of statistical significance was *p* < 0.05.

## 3. Results

**Figure 1:**
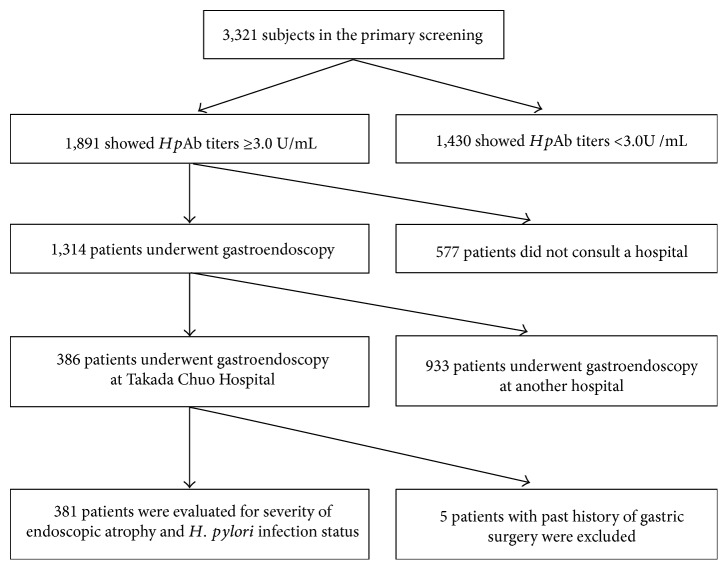
Patient flow. Among the 3321 primary screening subjects, 56.9% (1891/3321) showed *Hp*Ab ≥ 3.0 U/mL and 69.5% of these patients (1314/1891) could participate in secondary screening and underwent gastroendoscopy. On the other hand, 30.5% (577/1891) patients did not consult a hospital. Among 1314 patients who underwent gastroendoscopy, 386 patients consulted at Takada Chuo Hospital and underwent gastroendoscopy and 381 patients were able to evaluate severity of endoscopic atrophy and *Hp* infection status. On the other hand, 5 patients with past history of gastric surgery were excluded. In addition, 39 patients with past history of eradication therapy for *H. pylori* infection were included.

Among the 3321 primary screening subjects, 56.9% (1891/3321) showed *Hp*Ab ≥ 3.0 U/mL and 69.5% of these patients (1314/1891) could participate in the secondary screening and underwent gastroendoscopy (Figure 1). GC was detected in 10 patients (10/3321, incidence: 0.30%).

Table 1 shows the characteristics of patients in the groups divided based on the *Hp*Ab 10 U/mL cutoff. This division included 421 patients (32.0%) in the “negative-high” group and 893 patients (68.0%) in the “over-10 U/mL” group. Mean age and sex did not significantly differ between the two groups. GC was detected in 8 patients (0.9%) in the “over-10 U/mL” group; however, importantly, it was also detected in 2 patients (0.5%) in the “negative-high” group.

Table 2 shows details about the 10 patients in whom GC was detected. Both patients in the “negative-high” group with GC had O-3 atrophy. Overall, 8 (80%) had open-type atrophy. Nine (90%) of the patients had early GC.

Among the 1314 patients who underwent gastroendoscopy as a secondary screening, 381 patients who underwent gastroendoscopy at Takada Chuo Hospital were evaluated for severity of endoscopic atrophy and *Hp* infection status (Figure 1).

Table 3 shows the characteristics of the 381 patients evaluated for severity of endoscopic atrophy and *Hp* infection status. Among the 381 patients, 137 were in the “negative-high” group and 244 were in the “over-10 U/mL” group. Even in the “negative-high” group, 75.2% (103/137) of the patients had gastric atrophy.

22.6% of patients in the “negative-high” group also had a present infection. The rate of the past infection was significantly higher in the “negative-high” group (60.6%) than in the “over-10 U/mL” group (*p* < 0.0001). The combined rate of the present and past infection was 83.2% in the “negative-high” group.

**Figure 2:**
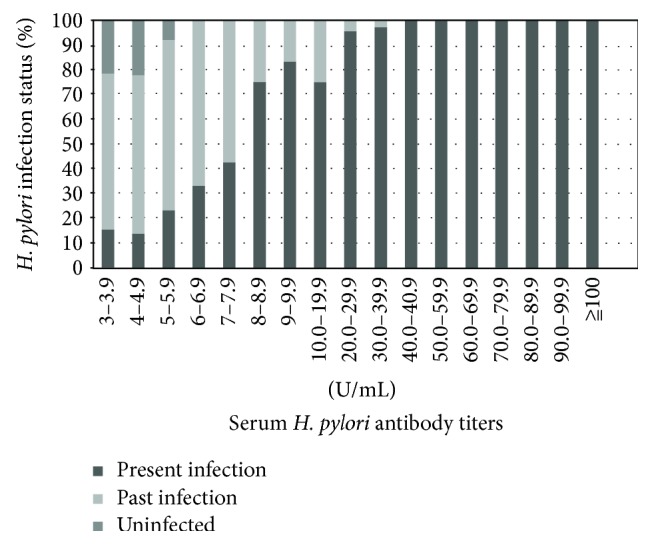
Relationship between *Hp*Ab titers and *Hp* infection status (*n* = 381). As the *Hp*Ab titers increased, the rate of present infection tended to increase, but conversely, the rate of past infection tended to decrease. Uninfected patients all had titers <6.0 U/mL.

Figure 2 shows the relationship between *Hp*Ab titers and *Hp* infection status. As the *Hp*Ab titers increased, the rate of the present infection tended to increase, but conversely, the rate of past infection tended to decrease.

## 4. Discussion

This study was the first prospective GC screening in residents of a rural city where *Hp*Ab titers ≥3.0 U/mL (i.e., more than the measurable limit) were defined as positive, and positive patients underwent endoscopy for a more detailed examination. In this GC screening study combining *Hp*Ab measurement and endoscopy, endoscopy in positive patients with *Hp*Ab ≥ 3.0 U/mL detected multiple GC cases. A total of 10 patients had GC (10/3321, incidence: 0.30%). Moreover, 90% (9/10) was early GCs, thus leading to be able to perform earlier treatment (Table 2).

In population-based GC screening in Japan, barium X-ray contrast studies are generally performed in asymptomatic persons age ≥ 40 years old; in those with suspected positive findings, endoscopy is recommended. However, the screening rates are very low, reaching only about 20% [27, 28] including Bungotakada city (11.1%: the mean screening rates of the previous 5-year period).

In this study, a participation rate could be improved probably due to our advertisement (including cable TV) and charge-free for the measurements.

As a result, we were able to discover GC cases more than the number by our previous X-ray contrast studies. The incidence of patients with GC that we could detect by population-based screening with X-ray contrast studies was 1.4 patients per year (i.e., 7 patients during the 5-year period in Bungotakada city) (our unpublished data). In contrast, we could detect 10 GC cases with only a one-year period of this study.

Endoscopy in positive patients with *Hp*Ab ≥ 3.0 U/mL enabled evaluation of GC risk factors such as endoscopic atrophy [2–7, 26] and diagnosis of *Hp* infection [9, 10]. During secondary screening, 63.4% (833/1314) of patients had atrophic gastritis and many patients with a present infection received eradication therapy. Of particular note is the fact that many patients in the “negative-high” group had a present or past *Hp* infection as a risk factor for GC (Figure 2) (Table 3). Endoscopy was required in these patients.

Uninfected persons in our study all had *Hp*Ab titers <6.0 U/mL, thus making it difficult to conclude that a cutoff value of 10 U/mL is optimal to assess *Hp* infection status or GC risk. Among the “negative-high” group, we found that the “negative-high” patients had GC risk. Therefore, if we used the cutoff value of 10 U/mL, the GC risk of these patients would be overlooked. In addition, these patients will not be able to follow up gastroendoscopy for the GC risk.

In addition, the overall mean age in our study was high, with a low screening rate among younger persons. If the screening rate in young generation increases, the eradication at an early stage is possible, and *Hp*-related GC may decrease later.

In the future, with an increase in the *Hp*-uninfected rate and further increase in eradication therapy, the definition of the “negative-high” group and interpretation of *Hp*Ab titers may need to be reconsidered.

In conclusion, using our screening criterion whereby *Hp*Ab titers ≥3.0 U/mL is defined as positive, endoscopy detected two cases of GC in a “negative-high” group that otherwise would be considered at low risk for GC. We could not deny the possibility that GC cases were included in subjects with serum *Hp*Ab titers less than 3 U/mL. We also could not determine the rates of the past or present infection among subjects with serum *Hp*Ab titers less than 3 U/mL. However, there are currently no ideal tests to accurately detect the infection status; therefore, we believe that our current screening system should be useful to detect GC cases effectively, and may decrease the number of *Hp*-related GC, followed by the decrease of the rate of GC death in the future.

We also found a correlation between *Hp*Ab titers and *Hp* infection status. “Negative-high” group has a risk of GC. Therefore, gastroendoscopy should be considered at least in patients with *Hp*Ab titers ≥3.0 U/mL on GC screening.

## Figures and Tables

**Table 1:** 

	“Negative high” group *n* = 421 (*Hp*Ab 3.0–9.9 U/mL)	“Over 10 U/mL” group *n* = 893 (*Hp*Ab ≧ 10 U/mL)	*p* value
Mean age	72 ± 9.7	70 ± 9.7	n.s.
Sex (male ratio)	36.0% (150)	36.0% (320)	n.s.
Gastric cancer	0.5% (2)	0.9% (8)	n.s.

*p* values were calculated by chi-square test. GC was detected in 8 patients in the “over-10 U/mL” group, but also in 2 patients in the “negative-high” group.

**Table 2:** Details about the 10 patients in whom GC was detected.

Age	Sex	Titers (U/mL)	Endoscopic atrophy	Therapy	Depth
78	M	5	O-3	Operation	SM
65	M	8	O-3	ESD	M
64	F	10	O-3	Operation	M
79	F	19	O-3	Operation	SE
71	M	22	O-2	ESD	M
77	M	24	O-3	Operation	SM
77	M	29	C-2	Operation	SM
75	F	30	O-2	Operation	M
64	M	58	O-1	Operation	M
46	M	≥100	C-3	ESD	M

**Table 3:** The characteristics of the 381 patients evaluated for severity of endoscopic atrophy and *Hp* infection status.

	“Negative high” group *n* = 137 (*Hp*Ab 3.0–9.9 U/mL)	“Over-10 U/mL” group *n* = 244 (*Hp*Ab ≧ 10 U/mL)	*p* value
Endoscopic atrophy (≧C-2)^※1^	75.2% (103)	96.3% (235)	*p* < 0.0001
Present infection	22.6% (31)	94.7% (231)	*p* < 0.0001
Past infection	60.6% (83)	5.3% (13)	*p* < 0.0001
Uninfected	16.8% (23)	0% (0)	*p* < 0.0001

*p* values were calculated by chi-square test. ^※1^The Kimura-Takemoto classification system even in the “negative-high” group, 75.2% (103/137) of the patients had ≥C-2 gastric atrophy. 22.6% of patients in the “negative-high” group had a present infection. The rate of past infection was significantly higher in the “negative-high” group (60.6%) than in the “over-10 U/mL” group (*p* < 0.0001). The combined rate of present and past infection was 83.2% in the “negative-high” group.

## References

[1] IARC Helicobacter pylori Working group (2014). *Helicobacter pylori* eradication as a strategy for preventing gastric cancer.

[2] Uemura N., Okamoto S., Yamamoto S. (2001). *Helicobacter pylori* infection and the development of gastric cancer. *The New England Journal of Medicine*.

[3] Asaka M., Kato M., Takahashi S. (2010). Guidelines for the management of *Helicobacter pylori* infection in Japan: 2009 revised edition. *Helicobacter*.

[4] Watabe H., Mitsushima T., Yamaji Y. (2005). Predicting the development of gastric cancer from combining *Helicobacter pylori* antibodies and serum pepsinogen status: a prospective endoscopic cohort study. *Gut*.

[5] Miki K. (2011). Gastric cancer screening by combined assay for serum anti-*Helicobacter pylori* IgG antibody and serum pepsinogen levels-“ABC method”. *Proceeding of the Japan Academy, Series B*.

[6] Mizuno S., Miki I., Ishida T. (2010). Prescreening of a high-risk group for gastric cancer by serologically determined *Helicobacter pylori* infection and atrophic gastritis. *Digestive Diseases and Sciences*.

[7] Nomura A., Stemmermann G. N., Chyou P.-H., Kato I., Perez-Perez G. I., Blaser M. J. (1991). *Helicobacter pylori* infection and gastric carcinoma among Japanese Americans in Hawaii. *The New England Journal of Medicine*.

[8] Watanabe Y., Kurata J. H., Mizuno S. (1997). *Helicobacter pylori* infection and gastric cancer: a nested case-control study in a rural area of Japan. *Digestive Diseases and Sciences*.

[9] Burucoa C., Delchier J. C., Courillon-Mallet A. (2013). Comparative evaluation of 29 commercial *Helicobacter pylori* serological kits. *Helicobacter*.

[10] Malfertheiner P., Megraud F., O’Morain C. (2007). Current concepts in the management of *Helicobacter pylori* infection: the Maastricht-3 consensus report. *Gut*.

[11] Cutler A. F., Prasad V. M. (1996). Long-term follow-up of *Helicobacter pylori* serology after successful eradication. *The American Journal of Gastroenterology*.

[12] Boda T., Ito M., Yoshihara M. (2014). Advanced method for evaluation of gastric cancer risk by serum markers: determination of true low-risk subjects for gastric neoplasm. *Helicobacter*.

[13] Salih B. A., Abasiyanik M. F., Saribasak H., Huten O., Sander E. (2005). A follow-up study on the effect of *Helicobacter pylori* eradication on the severity of gastric histology. *Digestive Diseases and Sciences*.

[14] Koizumi W., Tanabe S., Imaizumi H. (2003). Effect of anti-*Helicobacter pylori* IgG antibody titer following eradication of Helicobacter pylori infection. *Hepato-Gastroenterology*.

[15] Chey W. D., Wong B. C. (2007). American college of gastroenterology guideline on the management of *Helicobacter pylori* infection. *The American Journal of Gastroenterology*.

[16] Fock K. M., Talley N., Moayyedi P. (2008). Asia-Pacific consensus guidelines on gastric cancer prevention. *Journal of Gastroenterology and Hepatology*.

[17] Hamashima C., Shibuya D., Yamazaki H. (2008). The Japanese guidelines for gastric cancer screening. *Japanese Journal of Clinical Oncology*.

[18] Malfertheiner P., Megraud F., O’Morain C. (2017). Management of *Helicobacter pylori* infection: the Maastricht-5 consensus report. *Gut*.

[19] Matsuo T., Ito M., Takata S., Tanaka S., Yoshihara M., Chayama K. (2011). Low prevalence of *Helicobacter pylori*-negative gastric cancer among Japanese. *Helicobacter*.

[20] Kishikawa H., Kimura K., Ito A. (2015). Predictors of gastric neoplasia in cases negative for *Helicobacter pylori* antibody and with normal pepsinogen. *Anticancer Research*.

[21] Tatemichi M., Sasazuki S., Inoue M., Tsugane S. (2009). Clinical significance of IgG antibody titer against *Helicobacter pylori*. *Helicobacter*.

[22] Suzuki G., Cullings H., Fujiwara S. (2007). Low-positive antibody titer against *Helicobacter pylori* cytotoxin-associated gene a (CagA) may predict future gastric cancer better than simple seropositivity against H. Pylori CagA or against H. Pylori. *Cancer Epidemiology, Biomarkers & Prevention*.

[23] Yamaji Y., Mitsushima T., Ikuma H. (2002). Weak response of Helicobacter pylori antibody is high risk for gastric cancer: a cross-sectional study of 10,234 endoscoped Japanese. *Scandinavian Journal of Gastroenterology*.

[24] Kishikawa H., Nishida J., Ichikawa H. (2011). Fasting gastric pH of Japanese subjects stratified by IgG concentration against *Helicobacter pylori* and pepsinogen status. *Helicobacter*.

[25] The Japanese Society for Helicobacter Research (JSHR) Attention awakening from the Japanese Society for Helicobacter Research (JSHR) about positive, the negative judgment of the examination of serum *H. pylori* IgG antibody (in Japanese).

[26] Kimura K., Takemoto T. (1969). An endoscopic recognition of the atrophic border and its significance in chronic gastritis. *Endoscopy*.

[27] Itoh T., Saito M., Marugami N. (2015). Correlation between the ABC classification and radiological findings for assessing gastric cancer risk. *Japanese Journal of Radiology*.

[28] Gotoda T., Ishikawa H., Ohnishi H. (2015). Randomized controlled trial comparing gastric cancer screening by gastrointestinal X-ray with serology for *Helicobacter pylori* and pepsinogens followed by gastrointestinal endoscopy. *Gastric Cancer*.

